# Serine Protease Inhibitors Restrict Host Susceptibility to SARS-CoV-2 Infections

**DOI:** 10.1128/mbio.00892-22

**Published:** 2022-05-09

**Authors:** Ebba Rosendal, Ionut Sebastian Mihai, Miriam Becker, Debojyoti Das, Lars Frängsmyr, B. David Persson, Gregory D. Rankin, Remigius Gröning, Johan Trygg, Mattias Forsell, Johan Ankarklev, Anders Blomberg, Johan Henriksson, Anna K. Överby, Annasara Lenman

**Affiliations:** a Department of Clinical Microbiology, Umeå University, Umeå, Sweden; b Laboratory for Molecular Infection Medicine Sweden (MIMS), Umeå University, Umeå, Sweden; c Department of Molecular Biology, Umeå University, Umeå, Sweden; d Industrial Doctoral School, Umeå University, Umeå, Sweden; e National Clinical Research School in Chronic Inflammatory Diseases (NCRSCID), Karolinska Institutet, Solna, Sweden; f Institute for Experimental Virology, TWINCORE, Centre for Experimental and Clinical Infection Research, a joint venture between the Medical School Hannover and the Helmholtz Centre for Infection Research, Hannover, Germany; g Department of Biochemistry & Research Center for Emerging Infections and Zoonoses (RIZ), University of Veterinary Medicine Hannover, Hannover, Germany; h Wallenberg Centre for Molecular Medicine (WCMM), Umeå University, Umeå, Sweden; i Swedish National Veterinary Institute (SVA), Uppsala, Sweden; j Department of Public Health and Clinical Medicine, Section of Medicine, Umeå University, Umeå, Sweden; k Swedish Defence Research Agency, CBRN Defence and Security, Umeå, Sweden; l Department of Chemistry, Umeå University, Umeå, Sweden; m Sartorius Corporate Research, Umeå, Sweden; n Department of Molecular Biosciences, The Wenner-Gren Institute, Stockholm University, Stockholm, Sweden; o Microbial Single Cell Genomics Facility, SciLifeLab, Biomedical Center (BMC) Uppsala University, Uppsala, Sweden; Columbia University Medical College

**Keywords:** SARS-CoV-2, COVID-19, TMPRSS2, serpin, alpha-1-antitrypsin, A1AT, plasminogen activator inhibitor 1, PAI1, antithrombin III, ATIII

## Abstract

The coronavirus disease 2019, COVID-19, is a complex disease with a wide range of symptoms from asymptomatic infections to severe acute respiratory syndrome with lethal outcome. Individual factors such as age, sex, and comorbidities increase the risk for severe infections, but other aspects, such as genetic variations, are also likely to affect the susceptibility to SARS-CoV-2 infection and disease severity. Here, we used a human 3D lung cell model based on primary cells derived from multiple donors to identity host factors that regulate SARS-CoV-2 infection. With a transcriptomics-based approach, we found that less susceptible donors show a higher expression level of serine protease inhibitors SERPINA1, SERPINE1, and SERPINE2, identifying variation in cellular serpin levels as restricting host factors for SARS-CoV-2 infection. We pinpoint their antiviral mechanism of action to inhibition of the cellular serine protease, TMPRSS2, thereby preventing cleavage of the viral spike protein and TMPRSS2-mediated entry into the target cells. By means of single-cell RNA sequencing, we further locate the expression of the individual serpins to basal, ciliated, club, and goblet cells. Our results add to the importance of genetic variations as determinants for SARS-CoV-2 susceptibility and suggest that genetic deficiencies of cellular serpins might represent risk factors for severe COVID-19. Our study further highlights TMPRSS2 as a promising target for antiviral intervention and opens the door for the usage of locally administered serpins as a treatment against COVID-19.

## INTRODUCTION

On 11 March 2020, the coronavirus disease 2019 (COVID-19) was declared a pandemic by the World Health Organization, and now, 2 years later, the causative agent, severe acute respiratory syndrome coronavirus 2 (SARS-CoV-2), has claimed the lives of millions of people worldwide. COVID-19 is a complex disease with a wide spectrum of clinical manifestations. These range from asymptomatic or mild to moderate self-resolving respiratory syndromes to severe symptoms requiring hospitalization with potential lethal outcome (1). Several risk factors for severe disease have been identified, including old age and male sex, along with comorbidities such as diabetes, obesity, hypertension, cardiovascular disease, and chronic kidney disease (2–4). However, even young and otherwise healthy individuals can be severely affected and succumb to the infection. The reasons for the large variability of COVID-19 susceptibility and disease symptoms between individuals are largely unknown but suggest that individual genetic variations may influence the clinical outcome. Identification of host factors influencing SARS-CoV-2 infection would both improve our understanding of the viral pathogenesis and aid in the development of antivirals. Some genetic variations that influence SARS-CoV-2 susceptibility and disease severity have been identified in recent genome-wide association studies (GWAS) in large populations of COVID-19 patients, including differences in the ABO blood group system (5, 6), a gene cluster at locus 3p21.31 (including SLC6A20, LZTFL1, CCR9, FYCO1, CXCR6, and XCR1) (5, 7), a gene cluster that encodes antiviral restriction enzyme activators (OAS1, OAS2, and OAS3) (8, 9), and interferon signaling (IFNAR2) (8, 10).

SARS-CoV-2 is an enveloped positive-strand RNA virus that is transmitted between individuals mainly through respiratory droplets and direct contact. SARS-CoV-2 causes infection of the respiratory tract, where it infects epithelial cells through receptor-mediated endocytosis (11, 12). The receptor interaction and the subsequent entry of SARS-CoV-2 into cells is mediated by the protruding spike (S) protein. The S-protein consists of two subunits. The outer S1-subunit contains the receptor binding domain and mediates binding to the cellular receptor angiotensin-converting enzyme 2 (ACE2) (13), while the inner S2-subunit enables membrane fusion. To enable fusion, the spike protein requires proteolytic priming by cellular proteases at the S1/S2 and S2′ site. The first cleavage at the S1/S2 site occurs inside the cell upon biosynthesis and can be mediated by several cellular proteases, including furin (14, 15). The second proteolytic event at the S2′ site exposes the viral fusion peptide and can occur at the cell membrane by transmembrane serine protease 2 (TMPRSS2) or after endocytic uptake inside endosomes by cathepsin B and L (13). Orchestration of receptor binding and proteolytic cleavage of SARS-CoV-2 is essential for successful entry and infection; therefore, expression of ACE2 and TMPRSS2 is likely to dictate cell susceptibility and tropism (16). In line with this, it has also been suggested that ACE2 and TMPRSS2 DNA polymorphisms are associated with COVID-19 susceptibility and clinical outcome (17).

To extend our understanding of the underlying genetic factors that influence SARS-CoV-2 susceptibility, we have investigated donor-specific differences that regulate viral infection in primary lung cells. Primary bronchial epithelial cells from multiple donors were differentiated at an air-liquid interface (ALI) *in vitro* to resemble the human airway epithelium. These cells were subsequently infected with SARS-CoV-2, and infection rates as well as transcriptional changes were monitored. We found considerable donor-specific differences in infection rates, which were associated not with the antiviral innate immune response induced by viral infection, but rather, with the abundance of certain extracellular proteins. Among these extracellular proteins, we found several serine protease inhibitors (serpins) with higher expression in ALI-cultures from nonsusceptible donors. We validated the inhibitory function of these serpins during SARS-CoV-2 infection and showed their specific inhibition of TMPRSS2-mediated cleavage of the viral S-protein. Furthermore, we showed a distinct expression pattern of these serpins in primary lung cells and a cell type-specific upregulation upon infection. These results shed new light on the importance of respiratory protease/antiprotease balance as a susceptibility determinant for virus infections such as SARS-CoV-2.

## RESULTS

### Infection with low numbers of SARS-CoV-2 highlights donor differences in HBEC ALI-cultures.

**FIG 1:**
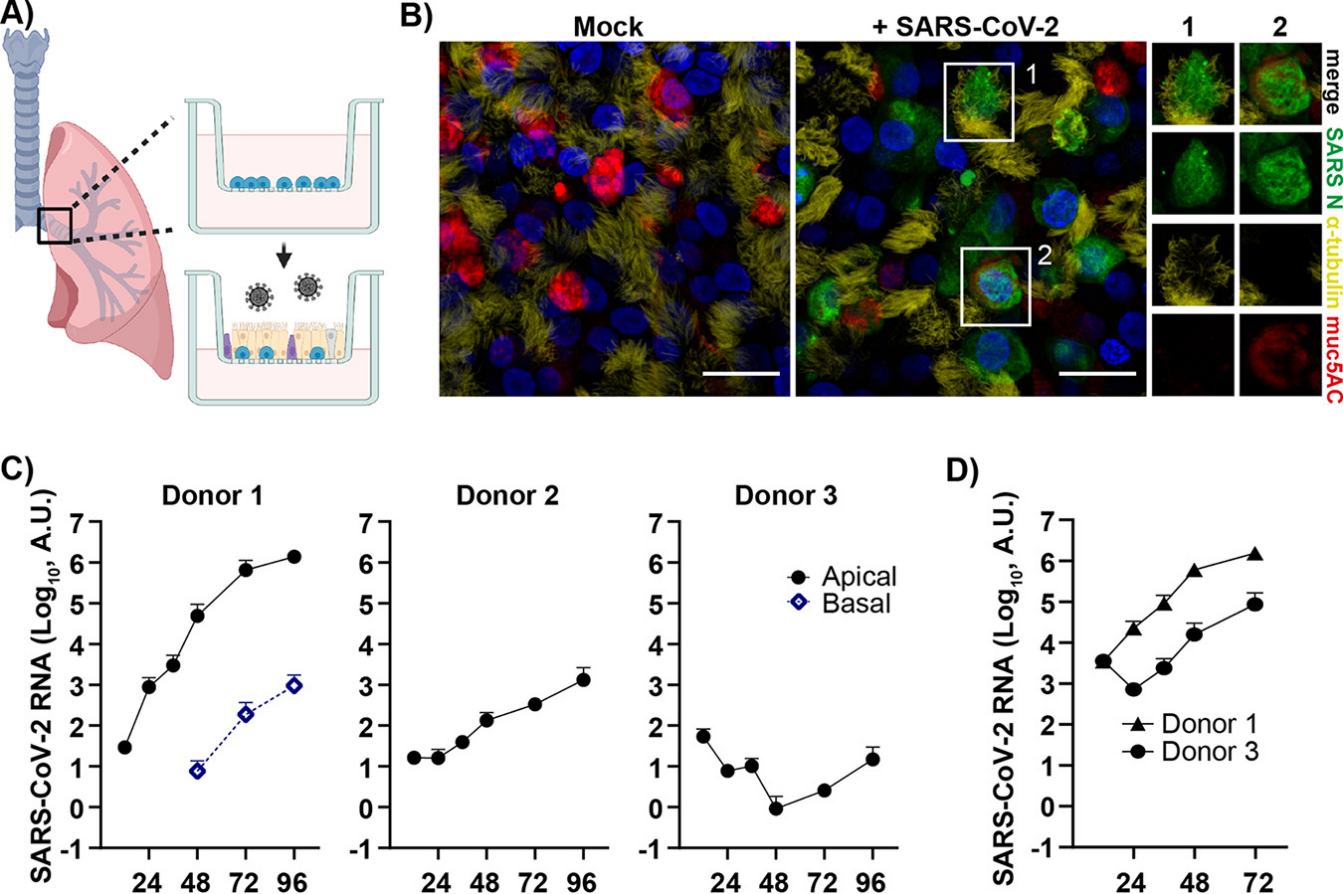
SARS-CoV-2 infection of human bronchial epithelial cells grown at an air-liquid interface identifies donor-dependent variation in infection levels. (A) Schematic of experimental design (created with BioRender.com). Air-liquid interface (ALI) cultures of human bronchial epithelial cells (HBECs) were generated, differentiated *in vitro*, and infected apically with SARS-CoV-2, and the infection was monitored over time. (B) HBEC ALI cultures fixed at 96 h postinfection were stained for viral nucleocapsid protein (NP), α-tubulin (ciliated cells), muc5AC (goblet cells), and DAPI (nuclei) and imaged at 60× on a confocal microscope. Scale bar = 20 μm. Infected cells showing costaining with either α-tubulin (1) or muc5AC (2) are shown in greater magnification. (C) Time kinetics of HBEC ALI cultures (*n* = 2) from three different donors (1, 2, and 3) infected with SARS-CoV-2 at an MOI of 0.05; (D) time kinetics for donor 1 and 3 at an MOI of 0.5. Infection was analyzed by qPCR of both apical (viral release during 1 h) and basolateral samples collected at the indicated time points.

To identify host factors influencing the susceptibility of different individuals to SARS-CoV-2 infections in a respiratory context, we used a model that closely resembles the human respiratory epithelium based on primary human bronchial epithelial cells (HBECs) isolated from different donors. These cells were differentiated (21 days) at an air-liquid interface (ALI) to form a pseudostratified, polarized epithelium containing an apical layer of fully functional secretory and ciliated cells and an underlying layer of basal cells (Fig. 1A and B). Immunofluorescent staining of the HBEC ALI cultures displayed the differentiated epithelium with ciliated cells (α-tubulin staining, yellow) and secretory goblet cells (muc5AC staining, red) (Fig. 1B). SARS-CoV-2 preferentially infected ciliated cells, but some infected goblet cells were also found (Fig. 1B). Initially, HBEC ALI cultures from three different donors were infected with SARS-CoV-2 at a multiplicity of infection (MOI) of 0.05. The course of infection was monitored by collection of basal media and apical secretions containing released progeny virus, and viral load was quantified by quantitative PCR (qPCR). A clear difference in infection levels was observed between the different donors (Fig. 1C). In donor 1, high numbers of released virions to the apical side were observed, increasing over time and corresponding to a well-established infection. This culture also displayed low virus shedding to the basal media at later time points. A moderate infection was observed in the HBEC ALI culture from donor 2, with no detectable virus in the basal media, indicating that the virus is preferentially shed to the apical side of the HBEC ALI cultures, and only highly infected cultures shed virus to the basal media. HBEC ALI cultures from donor 3 did not show an increase in viral release over time, indicating that no productive infection was established. To further investigate the susceptibility of donor 3, we infected the same cultures with a 10-fold higher MOI. With a higher MOI, the previously nonsusceptible cultures became infected; however, infection levels were still lower than for the more susceptible donor 1 (Fig. 1D). This shows that donor variations affect SARS-CoV-2 infection levels even at a high MOI, but infection with a smaller amount of virus will allow donor variations to have a more pronounced effect on the outcome of the infection.

### SARS-CoV-2 infection induce a strong innate immune response in highly infected HBEC ALI cultures.

**FIG 2:**
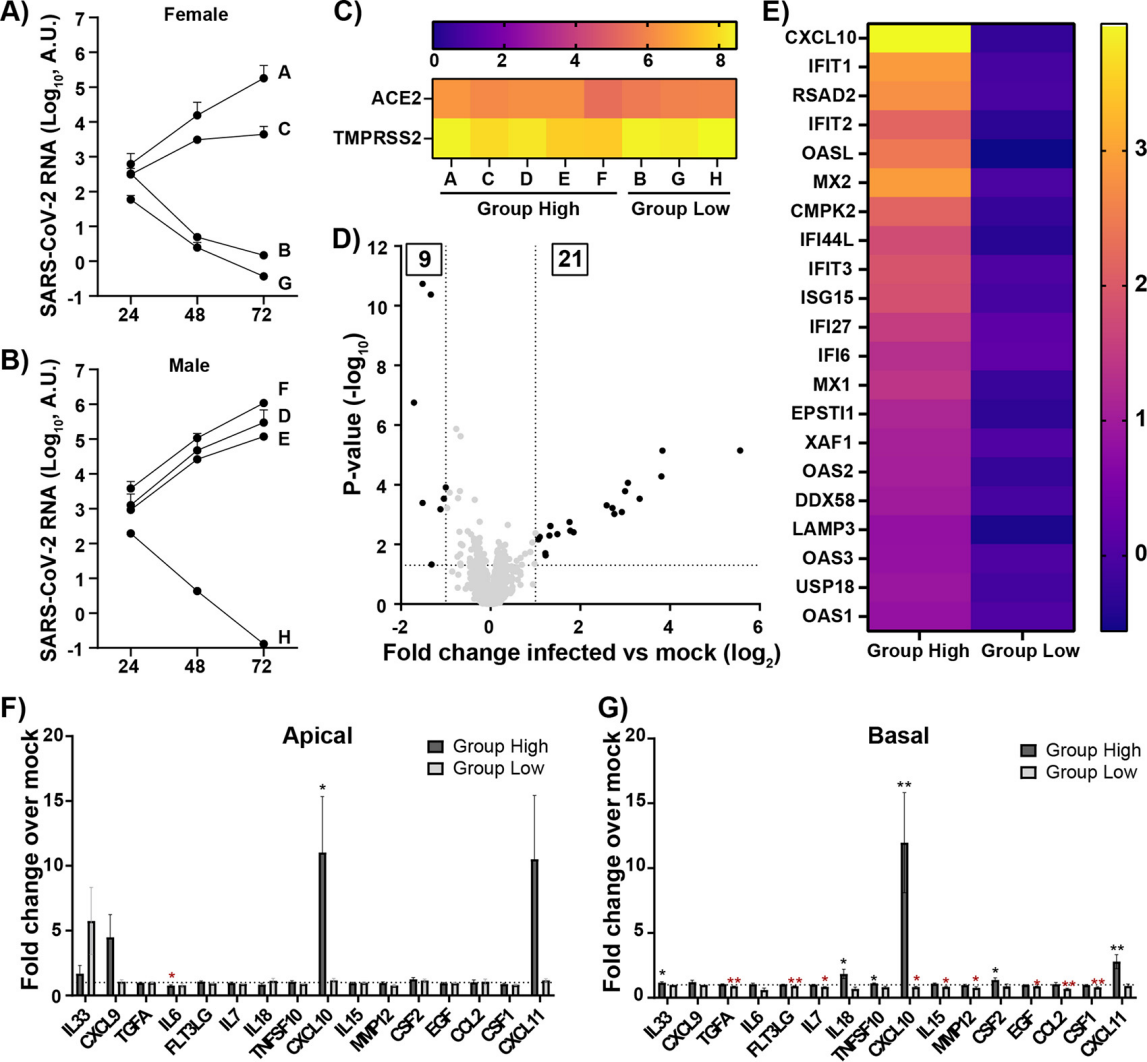
Donor differences in infection levels and immune response following SARS-CoV-2 infection of HBEC ALI cultures. HBEC ALI cultures from 8 different donors were infected with SARS-CoV-2 at an MOI of 0.05. The accumulated viral release from the apical side of cultures from (A) female donors, *n* = 4, and (B) male donors, *n* = 4, was quantified by qPCR at the indicated time points. (C) Heatmap displaying the mean expression (log_2_) of ACE2 and TMPRSS2 in HBEC ALI cultures from each donor. (D) Volcano plot showing differentially expressed genes between uninfected (mock) and infected HBEC ALI cultures in group high. The statistical *P* value (–log_10_) is plotted against the gene expression difference (log_2_). Dotted lines highlight the significance cutoff at log fold changes of −1/1 (vertical line) and at a *P* value of 0.05 (horizontal line). (E) Heatmap displaying the significantly upregulated genes in group high upon infection. Shown are the mean expression difference compared to individual mock samples for each group (log_2_ fold difference). (F and G) Cytokine levels in (F) apical and (G) basolateral samples collected at 72 h postinfection were analyzed using Proximity Extension Assay (Olink) and normalized to mock-treated samples for each donor individually. Mean values and standard error of the mean (SEM) are shown; statistical significance was calculated by unpaired *t* test (*, *P *< 0.05; **, *P *< 0.01).

With the possibility to study donor susceptibility to SARS-CoV-2 infection in the HBEC ALI cultures, we expanded our study to include HBECs from eight additional individuals, four females and four males. These HBEC ALI cultures were infected at day 21 of differentiation with a low MOI to allow differential infection between the donors. Apical samples were collected every day to monitor the course of infection. Time kinetic studies of apically released virus titers demonstrated a clear donor difference in SARS-CoV-2 infection levels, independent of donor gender (Fig. 2A and B). Based on their infection levels, the eight donors were divided into two groups, the highly to moderately infected group consisting of five donors (females A and C plus males D, E, and F), from here on denoted “group high,” and the noninfected group consisting of three donors (females B and G plus male H), from here on denoted “group low.” To enable identification of genes involved in regulating SARS-CoV-2 infection and genetic donor variance that could explain the difference in susceptibility, the HBEC ALI-cultures were harvested at 72 h postinfection and subjected to total RNA sequencing. The above-described grouping was confirmed by the RNA sequencing, with high levels of viral transcripts in the infected samples from group high and low or no viral transcripts in group low (Fig. S1A and B). Viral sequence comparison between the stock and the viral reads in the different donors showed only limited genetic drift in the donors (Table S1). Furthermore, a linear correlation was found between the levels of ORF10 in the HBEC ALI-cultures and the levels of apically released viral RNA as determined by qPCR, showing a strong connection between levels of cellular viral replication and released progeny viruses (Fig. S1C).

10.1128/mbio.00892-22.1 FIG S1 Levels of cellular viral transcripts determined by total RNA sequencing correlate with levels of viral RNA isolated from released progeny virus. (A and B) Level of viral transcripts in infected HBEC ALI cultures from (A) group high and (B) group low. (C) Correlation between accumulated viral release from the apical side of HBEC ALI cultures at 72 h postinfection with intracellular ORF10 normalized counts. Download FIG S1, TIF file, 1.0 MB. Copyright © 2022 Rosendal et al. 2022 Rosendal et al. https://creativecommons.org/licenses/by/4.0/ This content is distributed under the terms of the Creative Commons Attribution 4.0 International license.

10.1128/mbio.00892-22.5 TABLE S1 Point mutations in the SARS-CoV-2 genome after infection of HBEC ALI cultures. Download Table S1, DOCX file, 0.01 MB. Copyright © 2022 Rosendal et al. 2022 Rosendal et al. https://creativecommons.org/licenses/by/4.0/ This content is distributed under the terms of the Creative Commons Attribution 4.0 International license.

The observed variation in infection rate between cultures from different donors might depend on several factors, such as receptor and coreceptor abundance, innate immune response, or the nature of the respiratory mucosal barrier. To address this, we first analyzed the expression levels of the viral receptor ACE2 and the protease TMPRSS2, both known to be important for viral entry into host cells. No correlation was detected between the infection rate of the different donors and expression levels of ACE2 or TMPRSS2 (Fig. 2C). Next, we wanted to address potential differences in response to infection that could explain the observed donor variations. Statistical comparisons of genes expressed in SARS-CoV-2-infected HBEC ALI cultures from group high and group low against their corresponding uninfected controls identified a number of differentially expressed genes (DEGs) upon infection (Fig. 2D, Fig. S2A, Table S2). The significantly upregulated genes in group high were presented as a heatmap with increased expression values compared to uninfected controls for each group (Fig. 2E). Surprisingly, no genes were upregulated in group low, indicating that an efficient immune response clearing the viral infection is not the reason for the low infection levels seen in these cultures (Fig. S2A). Only a few genes were significantly downregulated in both group high and group low (Fig. S2B). Ingenuity pathway analysis of the 21 upregulated genes in group high showed a clear upstream regulation by interferon alpha, lambda, and gamma and highly activated canonical pathways involving interferon signaling and a role of hypercytokemia (Table S3). We also collected apical and basolateral samples from the infected HBEC ALI cultures at 72 h postinfection and analyzed these with a targeted cytokine panel based on Proximity Extension Assay, allowing quantitative concentration measurement of each cytokine (Fig. 2F and G). In correlation with the RNA transcriptomics data (Fig. 2D and E), we observed a large increase in CXCL10 upon infection on both the apical and basolateral side of cultures in group high but not in group low (Fig. 2F and G). Overall, few of the analyzed cytokines were differentially regulated in these cultures upon infection, and more changes in the secretion pattern were observed in the basal sample than in the apical sample. In addition to CXCL10, group high showed an induced expression of interleukin-33 (IL-33), IL-18, CXCL11, and CSF2 in the basal sample, whereas group low showed slightly reduced expression of transforming growth factor alpha (TGFA), Fms-related tyrosine kinase 3 ligand (FLT3GL), IL-15, matrix metallopeptidase 12 (MMP12), epidermal growth factor (EGF), C-C motif chemokine ligand 2 (CCL2), and colony-stimulating factor 1 (CSF1).

10.1128/mbio.00892-22.2 FIG S2 Differentially expressed genes in HBEC ALI cultures upon SARS-CoV-2 infection. (A) Volcano plot displaying differentially expressed genes between uninfected (mock) and infected HBEC ALI cultures in group low. The statistical *P* value (–log_10_) is plotted against the gene expression difference (log_2_). Dotted lines highlight the significance cutoff at log fold changes of −1/1 (vertical line) and at a *P* value of 0.05 (horizontal line). (B) Heatmap showing the significantly downregulated genes in group high and group low upon infection. Shown are the mean expression differences compared to individual mock samples for each group (log_2_ fold difference). Download FIG S2, TIF file, 0.4 MB. Copyright © 2022 Rosendal et al. 2022 Rosendal et al. https://creativecommons.org/licenses/by/4.0/ This content is distributed under the terms of the Creative Commons Attribution 4.0 International license.

10.1128/mbio.00892-22.6 TABLE S2 Differentially expressed genes upon infection in group high and group low compared against their respective uninfected controls. Download Table S2, XLSX file, 0.8 MB. Copyright © 2022 Rosendal et al. 2022 Rosendal et al. https://creativecommons.org/licenses/by/4.0/ This content is distributed under the terms of the Creative Commons Attribution 4.0 International license.

10.1128/mbio.00892-22.7 TABLE S3 Ingenuity pathway analysis of upregulated genes in group high upon infection. Download Table S3, XLSX file, 0.04 MB. Copyright © 2022 Rosendal et al. 2022 Rosendal et al. https://creativecommons.org/licenses/by/4.0/ This content is distributed under the terms of the Creative Commons Attribution 4.0 International license.

From these experiments, we conclude that the difference in infection levels of HBEC ALI cultures from different donors is dependent on neither expression levels of ACE2 and TMPRSS2, nor on the antiviral interferon response mounted by the cells upon infection, as group low showed a complete lack thereof.

### Antiviral activity in apical secretions explain donor differences.

**FIG 3:**
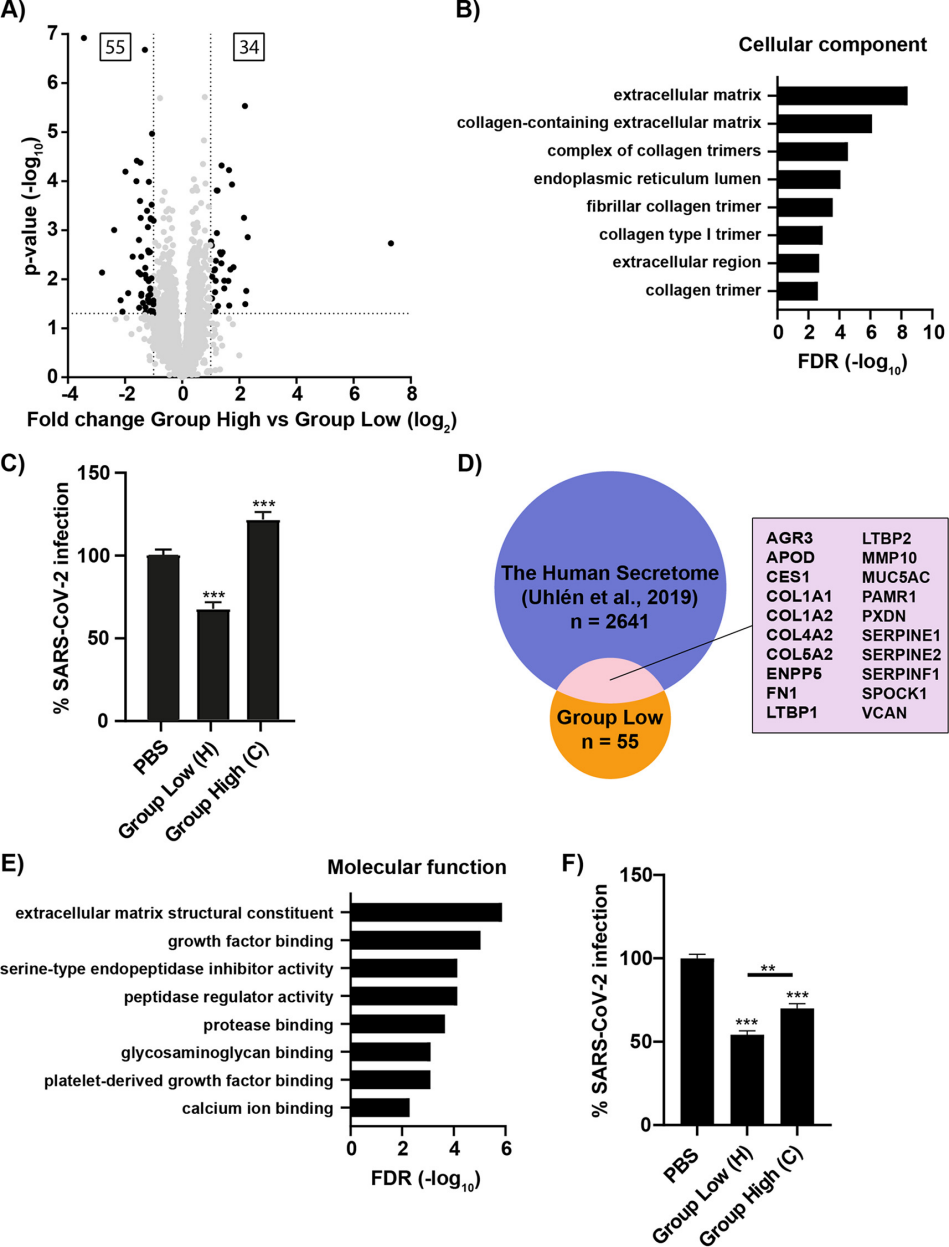
Identification of donor differences highlights secreted proteins as potential antiviral candidates in group low. (A) Volcano plot displaying differentially expressed genes between uninfected HBEC ALI cultures from group high versus group low. The statistical *P* value (–log_10_) is plotted against the gene expression difference (log_2_). Dotted lines highlight the significance cutoff at log fold changes of −1/1 (vertical line) and at a *P* value of 0.05 (horizontal line). (B) Gene Ontology term enrichment analysis showing the cellular components of the 55 upregulated genes in group low. The cellular components are plotted against their –log_10_ transformed false-discovery rate (FDR). (C) SARS-CoV-2 infection of Vero E6 cells 8 h postinfection following coincubation of cells with SARS-CoV-2 and apical secretion from donor C (group high) or donor H (group low). Infection is given as a percentage of PBS control; mean and SEM are shown, and statistical significance was calculated by unpaired *t* test (**, *P* < 0.01; ***, *P *< 0.001). (D) Venn diagram showing the overlap between human proteins known to be secreted, “the human secretome,” and the 55 upregulated genes in group low. The 20 overlapping genes are listed. (E) Gene Ontology term enrichment analysis displaying the molecular function of the 20 secreted proteins among the upregulated genes in group low. The molecular functions are plotted against their –log_10_ transformed false-discovery rate (FDR). (F) SARS-CoV-2 infection of Calu-3 cells 8 h postinfection following coincubation of cells with SARS-CoV-2 and apical secretion from donor C (group high) or donor H (group low). Infection is given as a percentage of PBS control; mean and SEM are shown; statistical significance was calculated by unpaired *t* test (**, *P* < 0.01; ***, *P *< 0.001).

In search for the genetic differences explaining the reduced susceptibility to SARS-CoV-2 infection in the HBEC ALI cultures of group low, we hypothesized that the difference between group high and group low might be in the basal gene expression of the cultures. Statistical comparison of the uninfected samples of group low versus those of group high identified 89 differentially expressed genes, with higher expression of 34 genes in group high and 55 genes in group low (Fig. 3A, Table S4). To focus on genes that could confer resistance to SARS-CoV-2 infection and identify commonalities between them, a gene ontology enrichment analysis was carried out on the 55 genes highly expressed in group low (Fig. 3B). Interestingly, the cellular component analysis showed a strong enrichment for extracellular proteins, especially extracellular matrix proteins. This suggested that the proteins involved in the reduced susceptibility to SARS-CoV-2 exert their function outside the cells, likely affecting the early steps of the viral life cycle. These results concur with the very low levels of viral transcripts and lack of immune response in the infected samples of group low (Fig. S1B), which indicates that a step prior to, or up to, viral replication is affected. To test this hypothesis, apical samples collected from uninfected HBEC ALI cultures of female C (group high) and male H (group low) were added to Vero E6 cells, followed by infection of SARS-CoV-2. The secretion from female C did not reduce the infection levels of SARS-CoV-2 but, rather, caused a modest increase (Fig. 3C). Interestingly, the secretion from male H showed a 40% reduction in infection. This indicate that the extracellular proteins from the HBEC ALI cultures may contain donor-dependent host factors that modulate SARS-CoV-2 infection. To identify extracellular proteins with potential antiviral activity, we performed a comparison between our list of 55 genes highly expressed in group low with the annotated human secretome (18) and identified 20 overlapping genes (Fig. 3D). These genes were further analyzed by gene ontology enrichment analysis for molecular functions. Among the most highly enriched functions, we found a group of extracellular matrix structural constituents, including collagens and mucins, and a group with serine-type endopeptidase inhibitory activity (Fig. 3E). The latter included a group of serine protease inhibitors, SERPINE1, SERPINE2, and SERPINF1, with the potential to inhibit the serine protease TMPRSS2 (19). As Vero E6 cells lack endogenous expression of TMPRSS2, we analyzed if the secretions from the group low and group high donors could affect SARS-CoV-2 infection of Calu3 cells. Interestingly, secretions from both donors reduced the infection in Calu3 cells, although secretions from group low reduced the infection to a greater extent (Fig. 3F). Taken together, these results indicate that the reduced susceptibility of cultures in group low may be a result of higher basal expression levels of secreted and extracellular proteins with antiviral activity.

10.1128/mbio.00892-22.8 TABLE S4 Differentially expressed genes in uninfected samples of group high against group low. Download Table S4, XLSX file, 0.5 MB. Copyright © 2022 Rosendal et al. 2022 Rosendal et al. https://creativecommons.org/licenses/by/4.0/ This content is distributed under the terms of the Creative Commons Attribution 4.0 International license.

### Extracellular SERPINS reduce SARS-CoV-2 infection through inhibition of TMPRSS2.

**FIG 4:**
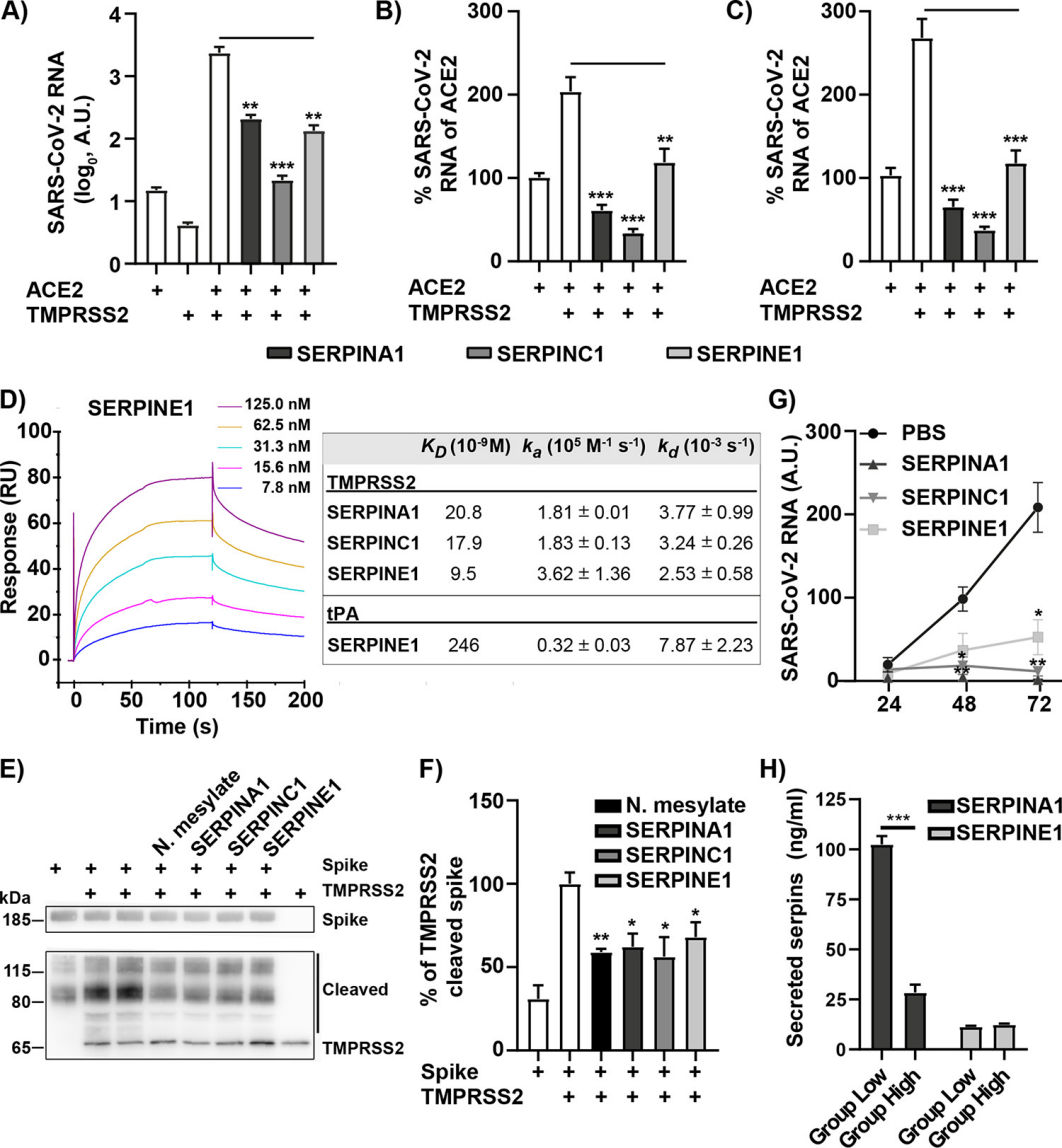
The investigated serpins reduce SARS-CoV-2 infection by inhibition of TMPRSS2-mediated spike protein cleavage. (A) HEK-293T cells transfected with the indicated expression plasmids for 24 h were infected with SARS-CoV-2 (MOI = 0.1) for 6 h, and viral RNA was measured by qPCR. (B and C) Posttransfection (24 h) HEK293T cells were infected for 2 h (MOI = 1) and then trypsinized, washed with PBS, and lysed, and the RNA was extracted. Levels of viral RNA were quantified from cDNA synthesized with (B) random hexamers (C) or only the forward primer selectively quantifying the negative sense RNA. Data are cumulative of three independent experiments performed in triplicate; mean and SEM are shown, and statistical significance was calculated by unpaired *t* test (*, *P *< 0.05; **, *P *< 0.01; ***, *P *< 0.001). (D) Surface plasmon resonance analysis of TMPRSS2 binding to individual serpins. A 2-fold dilution series of TMPRSS2 ranging from 125 nM down to 7.8 nM over immobilized SERPINE1 with results shown as response units (RU). Binding kinetics for all serpins are summarized to the right, including the natural target for SERPINE1, tissue plasminogen activator (tPA), as a positive control. (E) TMPRSS2-mediated S-protein cleavage in the presence or absence of individual serpins and the known protease inhibitor nafamostat mesylate. Data from three independent experiments were quantified, and a representative blot is shown. (F) The intensity of bands in panel E corresponding to cleaved S-protein was quantified using ImageJ (Fuji) and normalized to S-protein and TMPRSS2 control. Mean values and SEM are shown; statistical significance was calculated by unpaired *t* test (*, *P *< 0.05; **, *P *< 0.01). (G) HBEC ALI cultures were preincubated apically with recombinant SERPINE1, SERPINA1, or SERPINC1 and infected with SARS-CoV-2 at an MOI of 0.05. The accumulated viral release from the apical side was quantified by qPCR at the indicated time points (*n* = 3). Mean and SEM are shown; statistical significance was calculated by unpaired *t* test (*, *P *< 0.05; **, *P *< 0.01). (H) The concentrations of apically released SERPINA1 and SERPINE2 from HBEC ALI cultures from both group high and group low were determined by ELISA. The apical secretions were collected at three time points (*n* = 3). Mean and SEM are shown; statistical significance was calculated by unpaired *t* test (***, *P *< 0.001).

Since the cellular serine protease TMPRSS2 causes S-protein cleavage required for efficient entry and infection by SARS-CoV-2, it is possible that the presence of specific serpins in the HBEC ALI cultures of group low inhibits S-protein cleavage and thereby prevents infection. Among the upregulated genes in group low, four individual serpins were found (SERPINA1, SERPINE1, SERPINE2, and SERPINF1), although SERPINA1 had a slightly lower significance (*P *= 0.088) than the original cutoff (*P *= 0.05). First, we addressed if the reduction in infection in group low could be explained by the presence of these serpins. HEK-293T cells were made susceptible to SARS-CoV-2 infection by transfection with plasmids expressing ACE2 and TMPRSS2. These cells were also cotransfected with plasmids expressing individual serpins, followed by infection with SARS-CoV-2. We focused our attention on SERPINA1 and SERPINE1, as SERPINF1 lacks serine protease inhibitory activity and SERPINE1 and -E2 are paralogs. Additionally, SERPINC1, known as antithrombin III, is an FDA-approved anticoagulant and was included here due to its therapeutic potential against COVID-19 (ClinicalTrials.gov registration no. NCT04745442). To investigate the role of serpins during the first round of infection, a 6-h time frame was chosen, allowing replication, but not release, of progeny viruses and reinfection. All three serpins efficiently reduced infection of SARS-CoV-2 in HEK-293T cells as determined by quantification of viral RNA in cells 6 h postinfection (Fig. 4A). Next, the possible inhibition of SARS-CoV-2 entry by the serpins was analyzed. SARS-CoV-2 was added to HEK-293T cells coexpressing ACE2 and TMPRSS2 with or without individual serpins for 2 h followed by trypsination of the cells to remove bound but not internalized virus particles. A strong reduction in SARS-CoV-2 entry as measured by viral RNA could be observed with all three serpins (Fig. 4B). After viral membrane fusion, the viral genome is translated and negative-strand RNA synthesis is initiated. As TMPRSS2 cleavage of the spike is important for membrane fusion, we also specifically measured the synthesis of negative-stranded RNA in the presence and absence of the different serpins. A strong reduction of the negative-strand RNA to the level of ACE2-transfected cells was detected when coexpressing the serpins (Fig. 4C). This indicated that the antiviral effect of serpins occurs during the initial steps of the SARS-CoV-2 life cycle, preventing viral membrane fusion probably by inhibiting TMPRSS2-mediated cleavage of the viral S-protein. To investigate this further, we first performed surface plasmon resonance, allowing us to study the ability of these serpins to specifically interact with TMPRSS2, a prerequisite for inhibition. As a positive control, the natural target tissue plasminogen activator (tPA) was analyzed against SERPINE1. All three serpins showed a strong interaction with TMPRSS2 with nanomolar (nM) affinity (Fig. 4D). To ensure that this interaction leads to serpin-specific inhibition of TMPRSS2-mediated cleavage of the viral S-protein, an *in vitro* cleavage assay was performed. Recombinant TMPRSS2 and viral S-protein were incubated with or without recombinant SERPINA1, SERPINE1, and SERPINC1 protein or the positive control, nafamostat mesylate (20), followed by detection of SARS-CoV-2 S-protein and its cleavage products by Western blotting. In the presence of TMPRSS2, several cleavage products of the S-protein were detected, and these were reduced when adding the protease inhibitor nafamostat mesylate (Fig. 4E and F). All three serpins reduced the amount of cleavage product with a similar efficiency as the positive control, indicating that the serpins specifically inhibit TMPRSS2-mediated S-protein cleavage. Next, we wanted to see if the recombinant serpins could reduce SARS-CoV-2 infection of an HBEC ALI culture from group high. HBEC ALI cultures were pretreated apically with individual recombinant SERPINA1, SERPINE1, and SERPINC1 proteins, and the infection was monitored over time. All serpins tested reduced SARS-CoV-2 infection at 48 h and 72 h postinfection (Fig. 4G). Finally, as the expression of serpins in our HBEC ALI-cultures was based on transcriptional data (Fig. 3A, Table S4), we wanted to validate the presence of serpins in the apical secretions from these cultures. Apical samples were collected from uninfected HBEC ALI cultures of group high and group low, and the concentrations of SERPINA1 and SERPINE1 proteins were determined by enzyme-linked immunosorbent assay (ELISA). Although SERPINA1 was present in group high, the concentration of SERPINA1 was 4-fold higher in group low (Fig. 4H). The concentration of SERPINE1 was generally low, and no difference was observed between the two groups. These results indicate a larger antiviral contribution by SERPINA1 and support the antiviral effect of the apical secretions in Calu3 cells (Fig. 3F). The low levels of SERPINE1 in the apical secretions were rather unexpected, as the transcriptional difference between the two groups was high. From these data, we conclude that SERPINA1, -E1, and -C1 can prevent SARS-CoV-2 infection by specifically binding to and inhibiting TMPRSS2-mediated S-protein cleavage and subsequent entry and fusion of SARS-CoV-2 into target cells. However, the cellular distribution of the individual serpins and their relative antiviral effect against SARS-CoV-2 remain unclear.

### Single-cell RNA sequencing reveals distinct expression patterns of serpins among respiratory epithelial cells.

**FIG 5:**
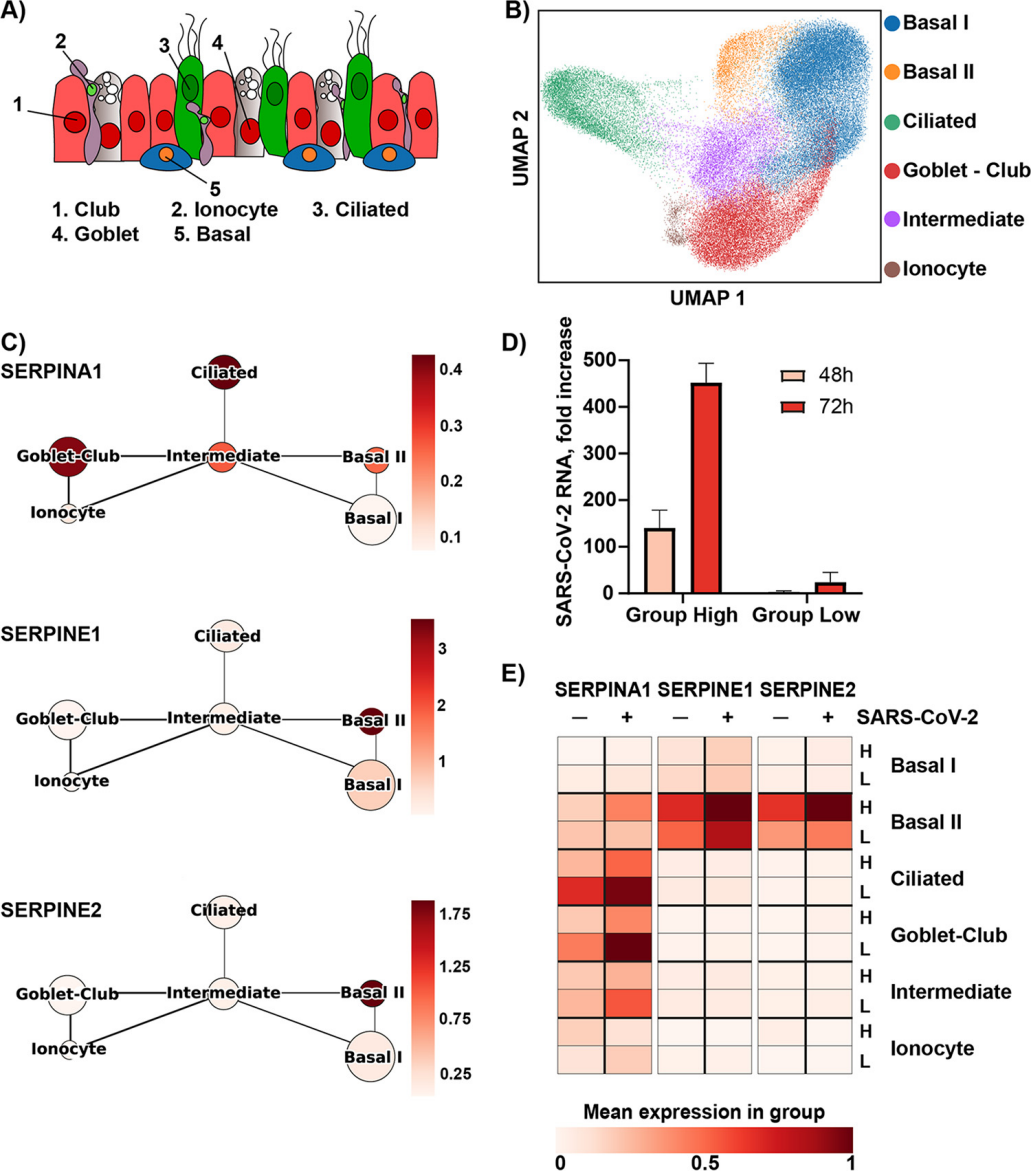
Single-cell RNA sequencing shows distinct expression patterns of serpins in primary lung cells. (A) Schematic illustration of cell types annotated in the single-cell RNA analysis of HBEC ALI cultures. (B) UMAP plot (bidimensional), colored by annotated cell clusters. (C) Coarse-grained graph showing mean cell cluster group expression for SERPINA1, SERPINE1, and SERPINE2. (D) Infection levels analyzed by qPCR and shown as fold increase over 12-h input. (E) Heatmap displaying the mean expression of SERPINA1, SERPINE1, and SERPINE2 upon SARS-CoV-2 infection (±, MOI = 0.05) in HBEC ALI cultures from a group high (H) and a group low (L) donor.

SERPINA1 and -E1 are known to be expressed in the lung and to be important for maintaining lung homeostasis (21), but their cellular expression patterns are poorly characterized. To better understand the antiviral role of each serpin during SARS-CoV-2 infection and deduce their cellular origin, we performed single-cell RNA-sequencing on HBEC ALI cultures from two donors. A routine single-cell analysis, including batch correction, was performed, and the data were visualized by uniform manifold approximation and projection (UMAP) nonlinear dimensionality reduction. With the use of cluster-driving genes and known lung epithelial cell markers (22), we found cells analogous to basal cells, ciliated cells, club cells, goblet cells, and ionocytes (Fig. 5A and B, Fig. S3A to D). Further cell types may be present but at such low numbers that they were not annotated. Two different clusters of basal cells were annotated based on their expression of the basal cell marker KRT14, referred to as *Basal I* (KRT14^low^) and *Basal II* (KRT14^high^), subpopulations of basal cells previously observed in human lung tissue (23). Furthermore, based on overlapping expression of club cell markers, such as SGCB1A1, and goblet cell markers, such as MUC5B/MUC5AC, a more general club-goblet cell annotation was used. Finally, a cluster of cells representing approximately 10% of the total number of cells sequenced appeared to be in an intermediate differentiation state and was annotated as intermediate. Quality control showed the clusters to be well mixed across time, donor, and infection state, suggesting that the described clustering primarily reflects cell type (Fig. S4A to C). Using this cell type annotation, we investigated the gene expression of SERPINA1, -E1, and -E2 among the different cell types (Fig. 5C). Interestingly, expression of SERPINE1 and -E2 was found almost exclusively in basal cells, specifically the KRT14^high^ cluster *Basal II*. SERPINA1 showed a broader expression pattern, with the highest level of expression found in ciliated cells and club-goblet cells, thereby supporting the higher concentration of SERPINA1 protein than that of SERPINE1 in the apical secretions of our HBEC ALI cultures (Fig. 4H and 5A). As it has previously been shown that the expression of SERPINs can be influenced by infection and inflammation (19, 24), we investigated how their expression in the different cell types was regulated upon SARS-CoV-2 infection. HBEC ALI cultures from two different donors, one from group low and one from group high, were infected with SARS-CoV-2 (MOI 0.5) or mock-treated. A high MOI was used to enable infection also of cultures from group low, and a productive infection was confirmed by the presence of high levels of ORF10 in various cell types, including ciliated, club-goblet, and basal cells (Fig. S4D). As seen previously, an overall higher infection level was observed in cultures from group high (Fig. 5D). Upon infection, a general increase of all three serpins was observed for both donors (Fig. 5E). Interestingly, the expression of the serpins remained limited to their respective cell types upon infection, suggesting a strong cell type-dependent expression pattern of these serpins in respiratory epithelial cells. Comparing the two donors, the group low donor had a higher expression of SERPINA1, especially in the ciliated cells and the club-goblet cells. SERPINE1 and E2 showed a relatively high expression in both donors, specifically in the *Basal II* cells, with slightly higher levels being found in the group high donor. Taken together, these results provide new insights to the expression pattern of serpins in the lung and their potential importance during SARS-CoV-2 infection.

10.1128/mbio.00892-22.3 FIG S3 Cell annotation of single-cell data. (A) Clustering of genes based on known marker genes. (B) Enriched genes in clusters according to Leiden. (C) UMAP showing clusters according to Leiden. (D) Pie chart reflecting global relative abundance and cell numbers of each annotated cell cluster. Download FIG S3, TIF file, 2.7 MB. Copyright © 2022 Rosendal et al. 2022 Rosendal et al. https://creativecommons.org/licenses/by/4.0/ This content is distributed under the terms of the Creative Commons Attribution 4.0 International license.

10.1128/mbio.00892-22.4 FIG S4 Quality control of single-cell data. (A to C) UMAP plot (bidimensional) colored by (A) time, (B) donor, and (C) infection status. (D) UMAP showing ORF10 distribution. Download FIG S4, JPG file, 1.3 MB. Copyright © 2022 Rosendal et al. 2022 Rosendal et al. https://creativecommons.org/licenses/by/4.0/ This content is distributed under the terms of the Creative Commons Attribution 4.0 International license.

## DISCUSSION

The wide range of COVID-19 clinical manifestations, along with the large number of individuals that develop severe COVID-19 without being associated with obvious risk factors, indicates that genetic differences in the population could play an important role for disease outcome. Here, we used a primary three-dimensional cell culture model of the human lung, originating from multiple donors, to identify genetic characteristics that contribute to SARS-CoV-2 susceptibility. We identified that nonsusceptible HBEC ALI cultures express extracellular proteins with antiviral activity that limited viral infection for at least 96 h. Among the extracellular proteins, serine protease inhibitors, serpins, were identified as key proteins, specifically inhibiting virus entry into cells by interfering with TMPRSS2-mediated cleavage of viral S-protein and viral fusion. This highlights variations in expression levels of cellular serpins as host factors influencing SARS-CoV-2 susceptibility.

Serpins belong to a class of suicide inhibitors that regulate several biological processes by inhibition of cellular proteases. We identified four serpins among the highly expressed genes in nonsusceptible HBEC ALI cultures, SERPINA1, SERPINE1, SERPINE2, and SERPINF1. All, except SERPINF1, display the common serine protease inhibitory activity, and we show here that both SERPINA1 (alpha-1 antitrypsin, A1AT) and SERPINE1 (plasminogen activator inhibitor, PAI-1) along with SERPINC1 (antithrombin III, ATIII) can reduce SARS-CoV-2 entry into target cells through inhibition of the serine protease TMPRSS2. To our knowledge, this is the first time that SERPINC1 has been shown to have substrate specificity for TMPRSS2 and display a direct antiviral activity against SARS-CoV-2. This ability was, however, recently shown for SERPINA1 in two separate publications, where A1AT (SERPINA1) was identified in a targeted approach to find TMPRSS2 inhibitors (25), and by a screen of peptides/proteins derived from bronchoalveolar lavage aiming to inhibit SARS-CoV-2 spike-driven entry (26). SERPINE1 has also been shown to inhibit TMPRSS2, and together with its ability to inhibit multiple cellular serine proteases, it was shown to have antiviral activity against influenza A virus (IAV) (19). Here, we connect the ability of SERPINE1 to inhibit TMPRSS2 with a reduction in SARS-CoV-2 entry and infection.

In support of our findings, population-based observations suggest that A1AT (SERPINA1) deficiency could be a potential risk factor for severe COVID-19 (27, 28). Similarly, PAI-1 (SERPINE1) knockout mice have been shown to be more susceptible to IAV infection, and partial PAI-1 deficiency, due to polymorphism in the human SERPINE1 gene, increased IAV susceptibility *in vitro* (19). A similar scenario is possible for SARS-CoV-2 infections and warrants further investigation. Although not identified in our data set, low levels of antithrombin III (SERPINC1) have been associated with increased mortality in COVID-19 (29). Whether this is due to a reduced ability to block viral entry leading to higher viral load or its natural involvement in regulation of the coagulation system remains unknown.

Both A1AT and antithrombin III deficiency are genetic disorders that can be treated with FDA-approved infusions of plasma-derived A1AT or plasma-derived/recombinant ATIII, respectively. Due to the suggested potential of A1AT (SERPINA1) to inhibit TMPRSS2-mediated SARS-CoV-2, entry along with its anticoagulatory and anti-inflammatory effects, A1AT therapy is currently undergoing clinical trials for treatment of COVID-19 (ClinicalTrials.gov registration no. NCT04385836, NCT04495101, and NCT04547140). Antithrombin III (SERPINC1) has also been included in clinical trials to combat SARS-CoV-2, but then only due to its role in the coagulation system (ClinicalTrials.gov registration no. NCT04745442). Our results show a direct inhibition of SARS-CoV-2 entry by ATIII, which could increase the potential beneficial effects of such treatment. However, since all these serpins play important roles in regulating biological pathways through inhibition of various proteases, treatment with these proteins requires caution. For the ability to prevent SARS-CoV-2 entry into target cells, a localized administration of the drug early in the infection would be beneficial and reduce the risk for side effects. Interestingly, both A1AT and ATIII have been shown to be biologically active following administration via inhalation in different model systems (30, 31). Another way to prevent TMPRSS2-mediated SARS-CoV-2 entry is by using chemical protease inhibitors, such as camostat mesylate or nafamostat mesylate (13, 20), also currently in clinical trials against COVID-19 (ClinicalTrials.gov registration no. NCT04418128 and NCT04473053). A benefit of using A1AT compared to low-molecular-weight protease inhibitors against COVID-19 is the primarily extracellular action of A1AT (32), which will only inhibit extracellular and plasma membrane-bound serine proteases, such as TMPRSS2, whereas inhibitors such as camostat mesylate can penetrate the cell, potentially increasing the risk of side effects.

Although all serpins identified here showed an antiviral activity against SARS-CoV-2, we found other secreted proteins to be upregulated in group low that may also affect SARS-CoV-2 infection. The apical secretions from the HBEC ALI cultures also displayed an antiviral effect in Vero E6 cells, indicating that additional antiviral mechanism can be of importance, as Vero E6 cells lack endogenous TMPRSS2 expression. One of the proteins that was upregulated in group low, muc5AC, a major gel-forming mucin expressed in the lungs, has previously been shown to protect against influenza infection in mice and in cell culture (33). Collagens were also overrepresented in group low, and the question if some of these proteins could affect SARS-CoV-2 infection warrants further investigation.

The use of primary HBEC ALI cultures far precedes the usage of cancerous cell lines that rarely retain the characteristics of the tissue from which they were derived. HBEC ALI cultures contain several important lung cell types that can be infected by SARS-CoV-2 and have enabled us to study donor-specific differences that would not have been possible otherwise. With single-cell RNA sequencing of the HBEC ALI cultures, we found a distinct expression pattern of the individual serpins, with SERPINA1 mainly found in the ciliated and club-goblet cells, and SERPINE1 and -E2 specifically found in KRT14^high^ basal cells (*Basal II*). Since ciliated and club-goblet cells are located at the apical surface, production and secretion of SERPINA1 from these cells *in vivo* would result in inhibition of TMPRSS2 at the natural site of infection. These results were confirmed by a high concentration of SERPINA1 in the apical secretions from the HBEC ALI cultures, compared to a relatively low concentration of SERPINE1. We also observed a higher concentration of SERPINA1 in the apical secretions of group low compared to those of group high, which correlated with their respective ability to reduce SARS-CoV-2 infection in Calu3 cells. The specific effect of SERPINE1 and -E2 expression from basal cells on viral infection is more difficult to anticipate and warrants further investigations. Upon infection of the HBEC ALI cultures from group high, a strong innate immune response was also detected. During productive infection, the cells responded with high expression levels of CXCL10, a major player in the recruitment of monocytes to the lung and a cardinal player in the cytokine storm of COVID-19 (34). Another cytokine secreted to the basolateral side in group high was IL-18, a member of the IL-1 family, which is a proinflammatory cytokine that has also been associated with the cytokine storm (35). A strong activation of the immune system followed by a cytokine storm is one of the largest clinical problems with COVID-19. However, if the viral infection is blocked already at the early stages of infection, such as entry, the initiation of the viral replication cycle is prevented and a productive infection cannot be established. Treatment options that target early steps will limit the overall viral burden in the patient and prevent virus-induced cell death that causes activation of the immune system that can result in the lethal cytokine storm.

Our results demonstrate an important role of individual cellular serpins in preventing TMPRSS2-cleavage of the S-protein and subsequent entry of SARS-CoV-2 into host cells and further suggest genetic variations in serpin expression as a potential determinant of SARS-CoV-2 susceptibility. These results also emphasize TMPRSS2 as a target for antiviral treatment and support the use of serpins for this purpose.

## MATERIALS AND METHODS

### Cell lines, virus, and reagents.

Vero E6 and HEK-293T cells were cultured in Dulbecco’s modified Eagle’s medium (DMEM, D5648 Sigma) supplemented with 5% fetal bovine serum (FBS) (HyClone), 100 U/mL penicillin, and 100 μg/mL streptomycin (PeSt, HyClone) at 37°C in 5% CO_2_. The clinical isolate SARS-CoV-2/01/human/2020/SWE (GenBank version no. MT093571.1) was kindly provided by the Public Health Agency of Sweden. The virus was propagated once in Vero E6 cells for 72 h and titrated by plaque assay. Sequencing of the viral stock was done by tiling PCR with Midnight primers and Oxford Nanopore Technologies (ONT) MinION sequencing. Nucleotide changes compared to the reference strain are summarized in Table S1. Recombinant SERPINE1 protein (PAI-1, 1786-PI), SERPINA1 protein (A1AT, 1268-PI), SERPINC1 protein (ATIII, 1267-PI), and nafamostat mesylate (3081) were purchased from R&D Systems. Active recombinant TMPRSS2 was purchased from Cusabio (CSB-YP023924HU). Tissue plasminogen activator (tPA, T0831) was purchased from Sigma. Expression plasmids encoding the precursor ectodomain of human SERPINE1 (SERPINE1-bio-His; Addgene plasmid no. 52077; http://n2t.net/addgene:52077; RRID:Addgene_52077), SERPINA1 (SERPINA-bio-His; Addgene plasmid no. 52182; http://n2t.net/addgene:52182; RRID:Addgene_52182), and SERPINC1 (SERPINC1-bio-His; Addgene plasmid no. 52076; http://n2t.net/addgene:52076; RRID:Addgene_52076) were a kind gift of Gavin Wright (36). Expression plasmids encoding human TMPRSS2 (pCSDest-HA-TMPRSS2; Addgene plasmid no. 154963; http://n2t.net/addgene:154963; RRID:Addgene_154963) was a kind gift from Utpal Pajvani, and expression plasmid encoding human ACE2 (hACE2; Addgene plasmid no. 1786; http://n2t.net/addgene:1786; RRID:Addgene_1786) was a kind gift from Hyeryun Choe (37).

### Generation of HBEC ALI cultures.

Primary human bronchial epithelial cells (HBEC) were cultured from proximal airway explants (38) obtained with informed consent from lung tissue from a total of 11 individual patients who underwent thoracic surgery at the University Hospital, Umeå, Sweden, in collaboration with Anders Blomberg (ethical permission approved by the Regional Swedish Ethical Review Authority in Umeå). HBEC were grown in bronchial epithelial cell medium (BEpiCM, SC3211-b, ScienCell) with recommended supplements (SC3262, ScienCell) and 100 U PeSt. For differentiation, 0.75 × 10^5^ cells were seeded onto a 6.5-mm semipermeable Transwell insert (0.4-μm pore polyester membrane insert, Corning) in differentiation medium (DMEM:BEpiCM 1:1 supplemented with 52 μg/mL bovine pituitary extract, 0.5 μg/mL hydrocortisone, 0.5 ng/mL human recombinant epidermal growth factor, 0.5 μg/mL epinephrine, 10 μg/mL transferrin, 5 μg/mL insulin, 50 nM retinoic acid [all from Sigma-Aldrich], and PeSt according to reference 39). Cells were maintained submerged for the first 7 days, after which the medium was removed from the apical side and cells were grown at the air-liquid interface for an additional 2 weeks to reach full differentiation. Medium was replaced three times per week with addition of fresh retinoic acid to the medium shortly before usage. Differentiation of the HBEC was assessed using light microscopy focusing on epithelial morphology, the presence of ciliated cells, and mucus production. The presence of ciliated cells and goblet cells was also determined with immunofluorescence (IF) using antibodies directed against acetylated tubulin and muc5AC, respectively, as described under “Immunofluorescence.”

### Virus infection of HBEC cultures.

**(i) Virus infection.** Immediately prior to infection, the apical side of the HBEC ALI cultures was rinsed three times with phosphate-buffered saline (PBS). For infection, 1.5 × 10^5^ PFU or 1.5 × 10^4^ PFU of SARS-CoV-2 in a total volume of 100 μL infection medium (DMEM/PeSt) was added to the apical compartment, corresponding to an approximate MOI of 0.5 and 0.05, respectively. Cells were incubated at 37°C and 5% CO_2_ for 2.5 h before the inoculum was removed and the cells washed with PBS to remove residual medium.

**(ii) Sample collection.** At the indicated time points, samples were collected from the apical and/or basolateral chamber. To quantify basolateral viral release, 100 μL of medium was collected from the basolateral chamber and immediately replaced with fresh medium. Furthermore, accumulated virus and mucus were collected from the apical side by the addition of 100 μL warm infection medium or PBS to the apical chamber, followed by a 1-h incubation at 37°C and 5% CO_2_. If infection medium was used for the collection, the apical chamber was subsequently washed once with PBS to remove residual medium.

**(iii) Treatment with recombinant serpin proteins.** 20 ng of recombinant serpin protein in 20 μL of PBS was added to the apical chamber of the HBEC ALI cultures 4 h prior to infection and replaced with freshly prepared protein at the point of infection, immediately after removal of the viral inoculum, and every 24 h during the course of infection.

### RNA extraction and reverser transcriptase quantitative PCR (RT-qPCR).

Viral RNA secreted from HBEC ALI cultures was extracted from 50 μL of sample using the QIAmp viral RNA kit (Qiagen), and cDNA was synthesized from a fixed volume of eluted RNA. Total RNA from cells was extracted using the Nucleo-Spin RNA II kit (Macherey-Nagel), and cDNA was synthesized from 500 ng of RNA using the high-capacity cDNA reverse transcription kit (Applied Biosystems). Actin was quantified using the QuantiTect primer assay (QT01680476, Qiagen) and the qPCRBIO SyGreen mix Hi-ROX (PCR Biosystems). SARS-CoV-2 was quantified using the qPCRBIO probe mix Hi-ROX (PCR Biosystems) and primers (forward, GTC ATG TGT GGC GGT TCA CT; reverse, CAA CAC TAT TAG CAT AAG CAG TTG T) and probe (CAG GTG GAA CCT CAT CAG GAG ATG C) specific for viral RdRp (Corman et al. [40]) on a StepOnePlus real-time PCR system (Applied Biosystems). For SARS-CoV-2, the probe was used according to Corman et al., whereas forward and reverse primers are modifications of Corman et al. made by Magnus Lindh at Sahlgrenska University Hospital, Gothenburg.

### Immunofluorescence.

HBEC ALI cultures were washed three times with PBS, apically and basally, prior to overnight fixation in 4% paraformaldehyde at 4°C. The fixed inserts were washed three times for 10 min with IF buffer (10 mM Na_2_HPO_4_/NaH_2_PO_4_ plus 130 mM NaCl plus 0.05% NaN_3_ plus 0.1% bovine serum albumin [BSA] plus 0.2% Triton X-100 plus 0.04% Tween 20, pH 7.4). Then, inserts were blocked for 1 h at room temperature in IF buffer plus 10% FBS and subsequently incubated with primary antibodies in IF buffer plus 10% FBS (SARS-CoV NP: 40143-R001, Sino Biological, 1:1000; mucin5AC: Ab-1 [45M1], no. MS-145-P, Thermo Fisher 1:200; acetylated tubulin: T6793-0.2 mL, Sigma, 1:200) overnight at 4°C. Before secondary antibody incubation, HBEC ALI inserts were washed as before, three times for 20 min, with IF buffer. Secondary antibodies and nuclear stain were diluted in IF buffer plus 10% FBS for 1 h at room temperature (AF488: Thermo Fisher A11088; AF568: Thermo Fisher A21144; AF647: Thermo Fisher A21240; 1:200) and Hoechst33342 (Thermo Fisher 62249; 1:10,000). For mounting, samples were washed three times with IF buffer for 20 min as described above and rinsed twice apically and basally for 5 min with PBS. A 4-mm punch (biopsy puncher, Miltex 33-34) from the insert was mounted on a cytoslide (Shandon, Thermo Fisher 5991057) on ProLong Gold antifade mounting medium (Thermo Fisher, P10144). Confocal images were acquired on a Leica SP8 confocal microscope with a 63× oil objective at the BICU imaging unit of Umeå University. Representative fields of view were imaged as z-stacks of 40 slices with 0.5-μm step size, and substacks of slices 1 to 20 were converted to maximum intensity projections using Fiji (41). Representative regions of interest were selected and presented as insets of a field of view.

### Bulk RNA sequencing.

At 72 h postinfection mock-infected (*n* = 1) and infected (MOI = 0.05, *n* = 2) HBEC ALI cultures from eight individual donors were washed three times with PBS, apically and basolaterally, before lysis and RNA extraction using the NucleoSpin RNA II kit (Macherey-Nagel) in accordance with the manufacturer’s instructions. RNA-seq library preparation was performed using Smart-seq2 (42), and the resulting libraries were sequenced on an Illumina NextSeq 500 system (75PE, v2.5; high-output flow cell). Reads were aligned using STAR 2.7.1a against a custom reference genome consisting of GRCh38 and Sars_cov_2.ASM985889v3. Gene expression was calculated using featureCounts (43), and differential expression analysis was performed using linear modeling with R and DESeq2 (44).

**Identification of SARS-CoV-2 genetic variants after infection of HBEC ALI cultures.** Variant call files were produced from the bulk RNA sequencing samples with the command bcftools mpileup -f combined_reference.fa example.bam | bcftools call -mv -Ob -o example.bcf. Variants related to isolate SARS-CoV-2/01/human/2020/SWE (GenBank version no. MT093571.1) were extracted using bcftools and grep and summarized with an R script (see “Data Availability”). Locations and amino acid substitutions were manually analyzed in Benchling using a common codon table.

### Cytokine quantification.

Samples from the apical and basolateral chambers of mock-infected (*n* = 3) and infected (MOI = 0.05, *n* = 3) HBEC ALI cultures from eight individual donors were collected at 72 h postinfection (hpi) and inactivated with Triton X-100 at a final concentration of 1%, followed by incubation at room temperature for 3 h. The levels of 45 cytokines were quantified using Proximity Extension Assay (PEA) technology, specifically the Olink Target 48 cytokine panel at Affinity Proteomics Uppsala (SciLifeLab Sweden, Uppsala University), which gives absolute (pg/mL) and relative (Normalized Protein Expression [NPX]) concentration measurements of 45 preselected cytokines.

### GO enrichment analysis.

The 55 upregulated genes from group low (Fig. 3A) were subjected to a Gene Ontology (GO) term enrichment analysis using the STRING database (45). The false-discovery rate (FDR) was –log_10_ transformed and plotted against the “cellular component” terms. The 20 extracellular proteins (Fig. 3D) were subjected to a GO analysis in a similar manner, and their “molecular function” was plotted against the –log_10_ transformed FDR.

### Effect of apical secretion from HBEC cultures on infection.

Apical secretion from uninfected HBEC ALI cultures at day 21 of differentiation was obtained by addition of 100 μL of PBS followed by 10 min of incubation at 37°C. The secretion was collected and frozen at −80°C until use. Apical secretion and SARS-CoV-2 (1,500 PFU) in PBS were mixed 1:1 and added to Vero E6 cells (3 × 10^4^ cells/well) seeded in 96-well plates (Greiner CELLSTAR) and incubated for 2 h at 37°C and 5% CO_2_. After 2 h, the inoculum was removed and replaced by 100 μL DMEM plus 2% FBS plus PeSt. Cells were fixed at 8 hpi in 4% formaldehyde, and the plates were washed with PBS, permeabilized (PBS plus 0.5% Triton X-100 plus 20 mM glycine) for 10 min at room temperature (RT) and subsequently stained with primary antibody for 1 h (SARS-CoV NP; Sino Biological 40143-R001; 1:1,000) followed by secondary antibody (A21206; Invitrogen; 1:1,000) for 30 min and DAPI (4′,6-diamidino-2-phenylindole) staining (0.1 μg/mL in PBS) for 5 min. The number of infected cells was quantified using a TROPHOS Plate RUNNER HD (TROPHOS SA, Marseille, France), and the infection was normalized against a PBS control.

### Early infection and entry assay.

HEK-293T seeded in 12-well format (1 × 10^5^ cells/well) was transfected with equal amounts of the indicated plasmids; pI.18-eGFP was used as a transfection control and filler to ensure a total of 1.2 μg of DNA per well. GeneJuice transfection reagent (Novagen) was used according to the manufacturer’s instructions. Then, 24 h posttransfection cells were washed once with PBS and infected for 1 h at 37°C in 5% CO_2_. The inoculum was replaced with DMEM plus 2% FBS plus PeSt. For the early infection experiment, the cells were washed with PBS and lysed for RNA extraction at 6 h postinfection (hpi). For the entry assay, 2 hpi, cells were detached with trypsin and washed three times with PBS before lysis and RNA extraction.

### Surface plasmon resonance.

CM5 sensor chips and an amine-coupling kit were purchased from Cytiva. All surface plasmon resonance (SPR) experiments were performed at 25°C in 10 mM phosphate buffer, pH 7.4, 140 mM NaCl, and 0.27 mM KCl running buffer. Data were collected with a Biacore T-200 instrument at a rate of 1 Hz. Serpin A1, serpin C1, and serpin E1 were coupled to the CM5 sensor chip by amine coupling reactions at pH 4 according to the manufacturer’s instructions to an immobilization density of 5,200, 2,700, and 3,300 response units (RU), respectively. The surface of the upstream flow cell was used as a reference and was subjected to the same coupling reaction but in the absence of protein. The TMPRSS2 analyte, or the positive control, tissue plasminogen activator, tPA, was serially diluted in running buffer in a 2-fold concentration dilution (125 nM to 7.81 nM for TMPRSS2, 1,000 nM to 1.95 nM for tPA) and then injected over the reference and experimental surfaces for 120 sec and a dissociation time of 120 sec at a flow rate of 30 μL/min. Blank samples containing only running buffer were also injected under the same conditions to allow for double referencing. After each cycle, the biosensor surface was regenerated with a 60-s pulse of 10 mM Tris-glycine (pH 1.5) at a flow rate of 30 μL/min.

### *In vitro* cleavage of SARS-CoV-2 spike protein.

**(i) SARS-CoV-2 spike protein production.** The plasmid encoding the 2019-nCoV S-protein was kindly provided by Jason McLellan, and the details of this construct have been previously described (46). Protein was produced using the Gibco ExpiCHO expression system (Thermo Fisher Scientific). ExpiCHO cells were transfected with the plasmid using ExpiFectamine reagent and OptiPRO SF medium in a 6 by 10^6^-cells/mL culture according to the manufacturer’s protocol and subsequently grown for 8 days at 37°C, 8% CO_2_, 80% humidity, and 100 rpm. ExpiCHO enhancer with ExpiCHO feed was added 1 day after transfection according to the manufacturer’s protocol; 8 days after transfection, the supernatant was cleared of cells and debris by centrifugation at 1,500 × *g*, 4°C, for 20 min and subsequently passaged through a 0.2- μM filter. The filtered supernatant was adjusted to pH 7.4, mixed with 3 mL of His-pure Ni-NTA resin (Thermo Fisher Scientific) in a 1-L Nalgene single-use PETG Erlenmeyer flask with baffled bottom (Thermo Fisher Scientific), and incubated at 4°C, 85 rpm, overnight. The resin with the attached protein was collected in an Econo-Column chromatography 2.5 by 20-cm column (Bio-Rad). The resin was washed with 50 mL of 20 mM imidazole/PBS, pH 7.4, and then eluted with 30 mL of 250 mM imidazole/PBS, pH 7.4. The resulting eluate was concentrated, and the buffer was exchanged to 50 mM Tris plus 250 mM NaCl, pH 8.0, using Amicon Ultra-15 centrifugal filters (100 kDa cutoff). Protein purity was determined by SDS-PAGE, and concentration was determined by a bicinchoninic acid (BCA) protein assay kit (Thermo Fisher Scientific). Protein was stored at a concentration of 1 mg/mL at −80°C until use.

**(ii) *In vitro* cleavage.** Recombinant TMPRSS2 was preincubated with individual recombinant serpin proteins 10 min prior to addition of spike protein at a final molar ratio of 1:3:4.5 (spike:TMPRSS2:serpin) followed by a 2-h incubation at 37°C, 300 rpm. The spike protein and its cleavage products were resolved on a 4 to 12% Bis-Tris denaturing gradient gel (NuPAGE, Invitrogen, Life Technologies) and transferred to a polyvinylidene difluoride (PVDF) membrane. The membrane was blocked with 5% milk in PBS-T (PBS plus 0.05% Tween 20) overnight at 4°C. Staining was carried out using a SARS-CoV-2 spike antibody (Novus Biologicals, NB100-56578) directed against the C terminus of the spike protein, enabling visualization of both intact spike and its cleavage products (1:1,000 dilution, in PBS-T plus 2.5% milk) followed by a 1:1,000 dilution of a horseradish peroxidase (HRP)-conjugated goat anti-rabbit IgG antibody (Novex, A16104) in PBS-T plus 2.5% milk. The protein bands were detected by chemiluminescence using Super Signal West Pico or Femto (Thermo Scientific) and visualized using the Amersham Imager 680. Pictures were taken every 10 s, and the relative cleavage efficiency was evaluated using ImageJ.

### Enzyme-linked immunosorbent assay (ELISA).

Apical secretions were collected from fully differentiated, uninfected HBEC ALI cultures by addition of 100 μL preheated PBS and incubation at 37°C for 10 min. Collected apical secretions were then serially diluted in PBS, and the concentration of SERPINA1 and SERPINE1 was determined using commercially available ELISA kits (Abcam, SERPINA1 ab108799, SERPINE1 ab108891) according to the instructions from the manufacturer.

### Single-cell RNA-seq of HBEC ALI cultures.

**(i) Sample preparation and library production.** Single-cell suspensions of HBEC ALI cultures were generated for untreated cells prior to infection (0 h) and for infected or mock-infected cells at 48 and 72 hpi. The apical and basolateral sides of the inserts were incubated with Dulbecco's Phosphate Buffered Saline (DPBS) (Gibco) plus 0.05% EDTA for 15 min at 37°C, followed by 3 subsequent washes. Cells were detached by addition of 100 μL TrypsinLE (Gibco) to the apical side followed by a 15-min incubation at 37°C. Dissociated cells were transferred to a tube containing FBS (HyClone) to inactivate the enzyme, and the procedure was repeated until all cells were detached. Cells were pelleted by centrifugation for 8 min at 300 × *g* and washed two times with cold DPBS to remove residual enzyme and FBS. Cells from the 48 and 72 hpi time points were fixed in accordance with the 10x Genomics methanol fixation protocol (https://support.10xgenomics.com/single-cell-gene-expression/sample-prep/doc/demonstrated-protocol-methanol-fixation-of-cells-for-single-cell-rna-sequencing) and stored at −80°C overnight. This enabled the samples to be transferred to the biosafety level 2 (BSL2) facility holding the 10x chromium controller. Libraries were prepared according to the manufacturer’s instruction using the 10x chromium controller (Chromium Single Cell 3′ protocol ver. 3.1). A total of 13,200 cells were loaded per well on a Chromium chip G, with a targeted recovery of 8,250 cells. The library was sequenced using NovaSeq 6000 instrument at National Genomics Infrastructure (NGI), SciLifeLab.

**(ii) Data processing and package information.** The 10x raw sequencing reads were aligned using Cell Ranger ver. 3.0.1 against a custom reference genome consisting of GRCh38 and Sars_cov_2.ASM985889v3. Vireo was used to infer the donor cell origins (47). The origin as either male or female was determined by the average abundance of X-inactive specific transcript (Xist) RNA. Gene counts were analyzed and processed using the Python ver. 3.6 packages SCANPY (ver. 1.5.2), umap (ver. 0.3.10), numpy (ver. 1.17.3), scipy (ver. 1.5.2), anndata (ver. 0.7.5), pandas (ver. 1.0.3), scikit-learn (ver. 0.23.2), statsmodels (ver. 0.11.1), python-igraph (ver. 0.8.2), louvain (ver. 0.7.0), and leidenalg (ver. 0.8.1). Cell outliers were filtered out based on the number of genes expressed (min_genes = 200), using scanpy.pp.filter_cells. Gene cell counts were normalized (sc.pp.normalize_total, target sum = 10^6^), and counts were pseudo-log transformed (sc.pp.log1p). Donor and single nucleotide polymorphism (SNP) information was assigned. Cell cycle gene information was annotated, scored, and regressed out of the raw data set. Doublet discrimination was performed using the Scrublet package (expected doublet rate cutoff = 0.03), and these cells were removed. SNP information was used to also filter out doublets. Furthermore, cells that could not be assigned to any donor were removed. Next, mitochondrial genes were annotated and scored (sc.pp.calculate_qc_metrics), and only cells containing less than 10% mitochondrial reads were kept for the analysis. Next, highly variable gene analysis was performed (sc.pp.highly_variable_genes), and mitochondrial and innate immunity genes were excluded.

**(iii) Batch integration and correction.** Scanorama was used to integrate the different data sets (scanorama.integrate_scanpy). Next, the integrated data set’s neighbor graph was computed (sc.pp.neighbors), and the BBKNN algorithm (48) was used for batch correction (bbknn.bbknn). Finally, the UMAP was computed using sc.tl.umap, with “n_components” = 2. The annotation was also informed by using 2 components, where clusters were better separated.

**(iv) Cell type annotation.** The Leiden algorithm (49) was used to compute a cell clustering graph (scanpy.tl.leiden), and genes were ranked per group using scanpy.tl.rank_genes_groups, specifying logistic regression as the method. The reason for this was that multivariate approaches have been suggested as more suitable for RNA-Seq data (50). Cell clusters were annotated manually, based on cluster-specific top-ranked gene and cell type-specific marker genes (22).

### Statistical analysis.

Statistical analysis was performed using Prism ver. 8 (GraphPad Software).

### Data availability.

The bulk and single-cell RNA-Seq gene counts have been deposited at ArrayExpress (accession no. E-MTAB-10687 and E-MTAB-10697). All the R and Python code has been deposited at https://github.com/henriksson-lab/covidali_r.git.
