# Supplementary figures and images for: Serine Protease Inhibitors Restrict Host Susceptibility to SARS-CoV-2 Infections
Source: mBio. 2022 May 9;13(3):e00892-22. doi: 10.1128/mbio.00892-22 (PMC9239148; doi:10.1128/mbio.00892-22)

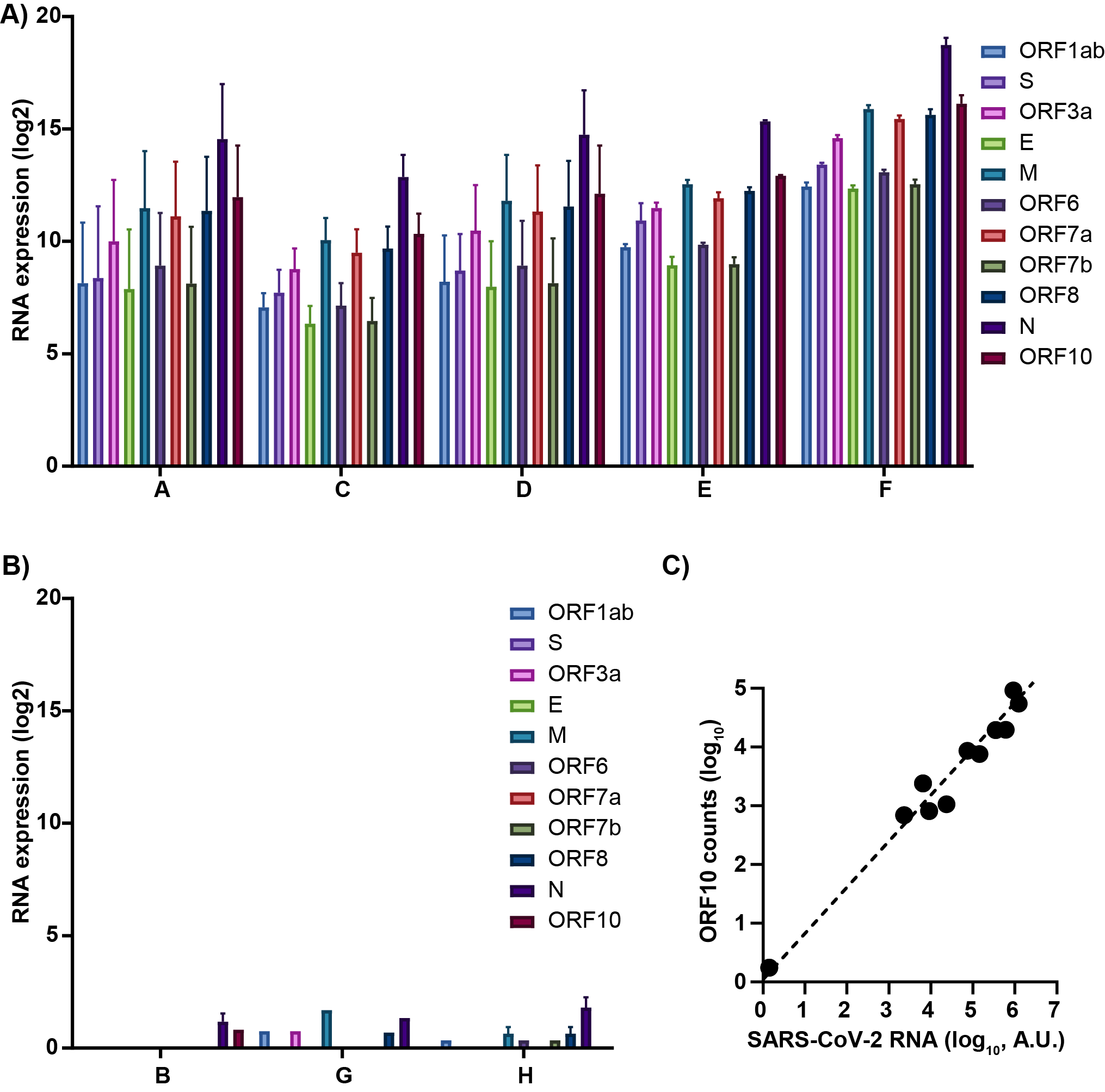

Supplement: FIG S1 [file mbio.00892-22-s0005.tif]

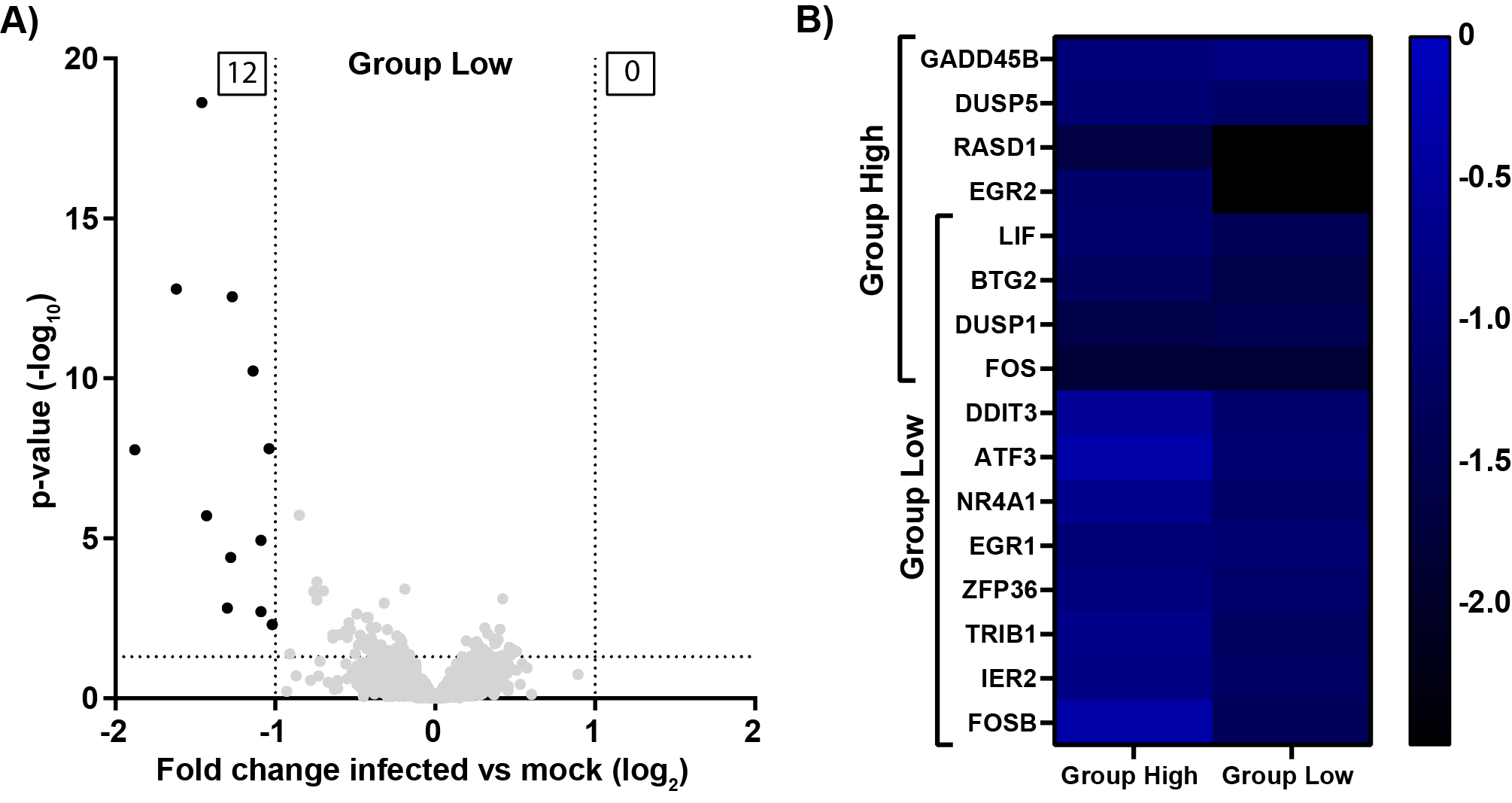

Supplement: FIG S2 [file mbio.00892-22-s0006.tif]

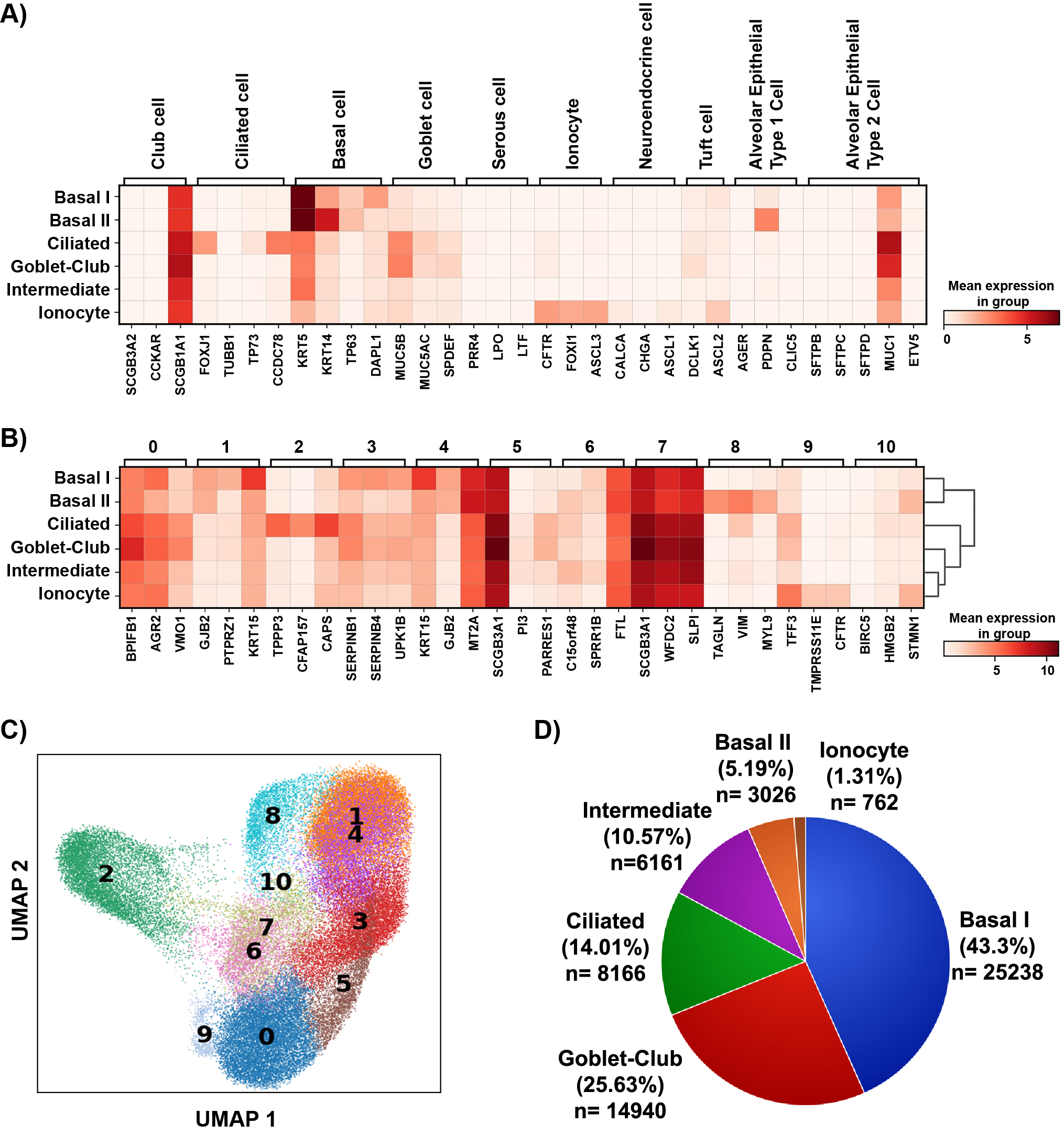

Supplement: FIG S3 [file mbio.00892-22-s0007.tif]

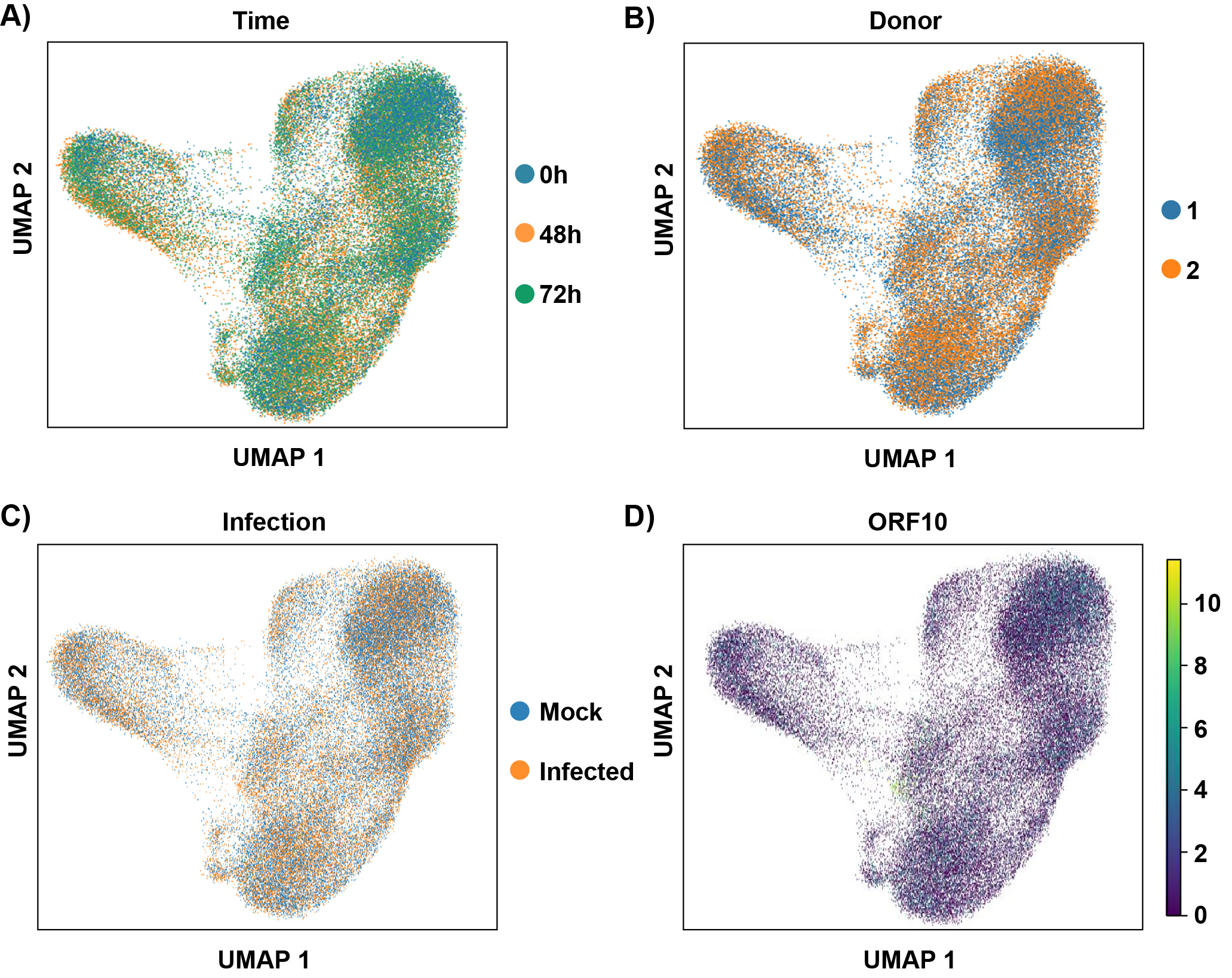

Supplement: FIG S4 [file mbio.00892-22-s0008.jpg]
