# Supplementary material for: Serine Protease Inhibitors Restrict Host Susceptibility to SARS-CoV-2 Infections
Source: mBio. 2022 May 9;13(3):e00892-22. doi: 10.1128/mbio.00892-22 (PMC9239148; doi:10.1128/mbio.00892-22)
Supplement: TABLE S1 [file mbio.00892-22-s0001.docx]

| Nucleotide position | Nucleotide in FHM reference, MT093571.1 | Nucleotide in viral stock | Nucleotide in  HBEC ALI-cultures | Mutation present in virus stock before HBEC ALI | Location | Amino acid exchange |
| --- | --- | --- | --- | --- | --- | --- |
| 2717 | A | G* | G* | yes | orf1ab polyprotein, nsp2 | Ser (S) 🡪 Gly (G) |
| 13225 | G | C* | C* | yes | orf1ab polyprotein, nsp10 | Ser (S) 🡪 Ser (S) |
| 13226 | C | T* | T* | yes | orf1ab polyprotein, nsp10 | Leu (L) 🡪 Phe (F) |
| 22100 | G | N | A |  | S | Glu (E) 🡪 Lys (K) |
| 23952 | G | T* | T* | yes | S | Cys (C) 🡪 Phe (F) |
| 25381 | A | A* | C |  | S | Thr (T) 🡪 Thr (T) |

*Nucleotide same as in isolate Wuhan-Hu-1, MN908947.3
